# Akt1-associated actomyosin remodelling is required for nuclear lamina dispersal and nuclear shrinkage in epidermal terminal differentiation

**DOI:** 10.1038/s41418-020-00712-9

**Published:** 2021-01-18

**Authors:** Clare Rogerson, Duncan J. Wotherspoon, Cristina Tommasi, Robert W. Button, Ryan F. L. O’Shaughnessy

**Affiliations:** 1 Centre for Cell Biology and Cutaneous Research, Blizard Institute, Barts and The London School of Medicine and Dentistry, Queen Mary University of London, London, UK; 2 Immunobiology and Dermatology, UCL Great Ormond Street Institute of Child Health, London, UK; 3 Present Address: School of Cellular & Molecular Medicine, University of Bristol, Bristol, UK

**Keywords:** Cell biology, Physiology

## Abstract

Keratinocyte cornification and epidermal barrier formation are tightly controlled processes, which require complete degradation of intracellular organelles, including removal of keratinocyte nuclei. Keratinocyte nuclear destruction requires Akt1-dependent phosphorylation and degradation of the nuclear lamina protein, Lamin A/C, essential for nuclear integrity. However, the molecular mechanisms that result in complete nuclear removal and their regulation are not well defined. Post-confluent cultures of rat epidermal keratinocytes (REKs) undergo spontaneous and complete differentiation, allowing visualisation and perturbation of the differentiation process in vitro. We demonstrate that there is dispersal of phosphorylated Lamin A/C to structures throughout the cytoplasm in differentiating keratinocytes. We show that the dispersal of phosphorylated Lamin A/C is Akt1-dependent and these structures are specific for the removal of Lamin A/C from the nuclear lamina; nuclear contents and Lamin B were not present in these structures. Immunoprecipitation identified a group of functionally related Akt1 target proteins involved in Lamin A/C dispersal, including actin, which forms cytoskeletal microfilaments, Arp3, required for actin filament nucleation, and Myh9, a component of myosin IIa, a molecular motor that can translocate along actin filaments. Disruption of actin filament polymerisation, nucleation or myosin IIa activity prevented formation and dispersal of cytoplasmic Lamin A/C structures. Live imaging of keratinocytes expressing fluorescently tagged nuclear proteins showed a nuclear volume reduction step taking less than 40 min precedes final nuclear destruction. Preventing Akt1-dependent Lamin A/C phosphorylation and disrupting cytoskeletal Akt1-associated proteins prevented nuclear volume reduction. We propose keratinocyte nuclear destruction and differentiation requires myosin II activity and the actin cytoskeleton for two intermediate processes: Lamin A/C dispersal and rapid nuclear volume reduction.

## Introduction

The mammalian epidermis is an essential barrier between an organism and its environment [1, 2]. The epidermis has four layers of keratinocytes: basal, spinous, granular and cornified, the last of which is comprised of corneocytes [3, 4]. Formation of a healthy skin barrier requires continual differentiation of epidermal keratinocytes into corneocytes [4]. Transition of keratinocytes from granular layer cells to corneocytes is a highly controlled and irreversible terminal process. As part of this, keratinocytes undergo major morphological changes, involving removal of all their intracellular organelles, including nuclei, allowing corneocytes to contain an extremely high proportion of keratin and form the rigid outer epidermal cell layer [4, 5].

Disruption of nuclear degradation leading to retention of nuclei in the cornified layer, known as parakeratosis, is a frequent observation in many skin diseases [6, 7]; however, the mechanisms by which keratinocytes remove their nuclei are not well defined [4, 8]. Nuclear removal is dependent on Akt1 kinase. Akt1 shRNA knockdown decreased phosphorylation of the nuclear lamina component Lamin A/C, decreased Lamin A/C degradation, and increased nuclear retention in the cornified layer [9, 10]. Subsequent to nuclear lamina breakdown, DNA degrading enzymes are required for nuclear breakdown; murine epidermis lacking DNase1L2 and DNase2 is parakeratotic, however the parakeratotic nuclei lack Lamin A/C indicating DNA degradation occurs after lamina removal [11]. Proteins important in autophagic processes are also required for removal of nuclear content; dysregulation of autophagy-related proteins correlates with decreased nuclear degradation and the autophagic marker LC3 localises close to the nucleus in differentiating cells [12, 13].

The transition from granular keratinocytes to corneocytes occurs over a 24 h period, with the nuclear removal process estimated to take 6 h [4, 14]. However, the mechanisms preceding removal of the nucleus are currently unidentified [8]. Using rat epidermal keratinocytes (REKs), which spontaneously differentiate in submerged culture, we have followed nuclear degradation in an in vitro model without forced initiation of differentiation by calcium switch. We identified Akt1-dependent dispersion of nuclear lamina components throughout the cytoplasm in differentiating keratinocytes that precedes nuclear degradation. Following fluorescently tagged nuclear proteins in real time has allowed us to identify rapid nuclear shrinkage as an important nuclear degradation intermediate. We also isolated a ‘degradosome’ complex of proteins that associate with Akt1, including actin and the actin-binding proteins Myh9 and Arp3 that are involved in nuclear removal. These results provide evidence for two nuclear degradation intermediates in keratinocyte differentiation and the characterisation of a protein complex important for nuclear degradation regulation.

## Materials and methods

### Antibodies and materials

**Fig. 1:**
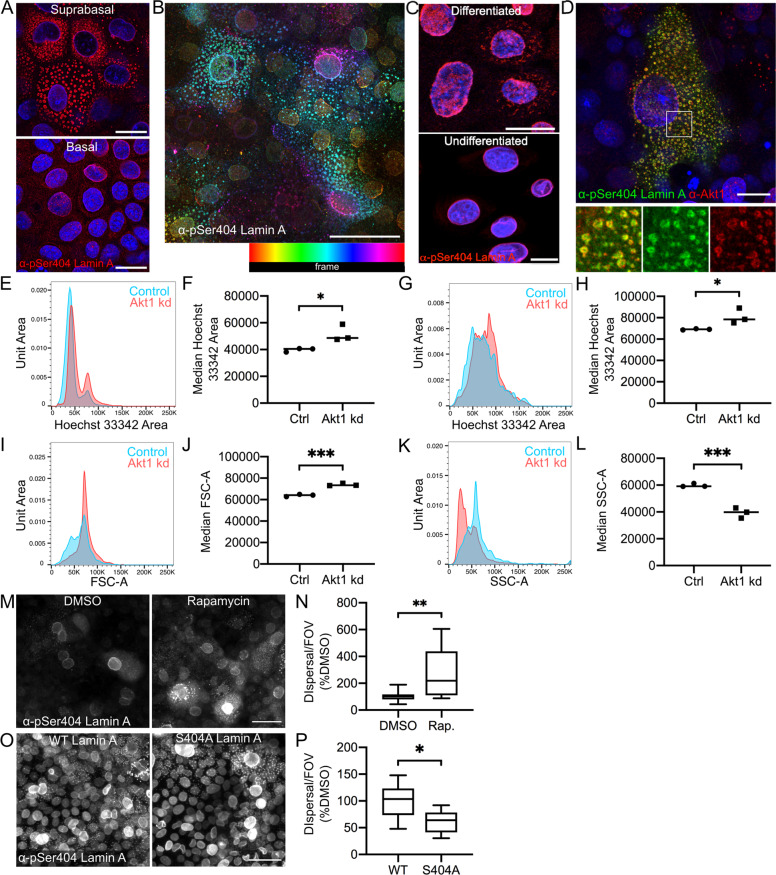
Phosphorylated Lamin A in keratinocyte differentiation and nuclear degradation. **A** pSer404 Lamin A/C staining in single confocal sections of basal and suprabasal REKs. Scale bars = 20 µm. **B** pSer404 Lamin A/C signal colour-coded according to the frame of the z-stack. Yellow = basal signal, blue and pink = more suprabasal signal. Scale bar = 20 µm. **C** pSer404 Lamin A/C staining in single confocal sections of undifferentiated and calcium switched differentiated human keratinocytes. Scale bars = 20 µm. **D** Akt1 co-localises with pSer404 Lamin A/C in a single confocal section of suprabasal REKs. Box indicates enlarged area, scale bar = 20 µm. **E** Representative area of Hoechst 33342 staining (nuclear content) of control or Akt1 knockdown (kd) REKs. **F** Median area of Hoechst 33342 staining (nuclear content) of control or Akt1 knockdown REKs, in triplicate *p ≤ 0.05. **G** Representative area of Hoechst 33342 staining of control or Akt1 knockdown large differentiating REKs, gated on high FSC and SSC. **H** Median area of Hoechst 33342 staining of control or Akt1 knockdown large differentiating REKs, in triplicate *p ≤ 0.05. **I** Representative FSC-A (Area) area of control or Akt1 knockdown large differentiating REKs. **J** Median FSC-A in control or Akt1 knockdown large differentiating REKs, in triplicate ***p ≤ 0.001. **K** Representative SSC-A (Area) of control or Akt1 knockdown large differentiating REKs. **L** Median SSC-A in control or Akt1 knockdown large differentiating REKs, in triplicate ***p ≤ 0.001. **M** DMSO or Rapamycin treated post-confluent REKs stained for pSer404 Lamin A/C. Maximum projections of confocal z-stacks, scale bar = 50 µm. **N** Number of cells with dispersed pSer404 Lamin A/C per field of view (FOV), 2 independent experiments, ≥4 FOV, Welch’s *t* test, **p ≤ 0.01. **O** REKs expressing WT or S404A Lamin A stained for pSer404 Lamin A/C. Epifluorescence image, scale bar = 50 µm. **P** Number of cells with dispersed pSer404 Lamin A/C per FOV compared to WT Lamin A. Two independent experiments, three FOV per experiment, Welch’s *t*-test, *p ≤ 0.05.

**Fig. 2:**
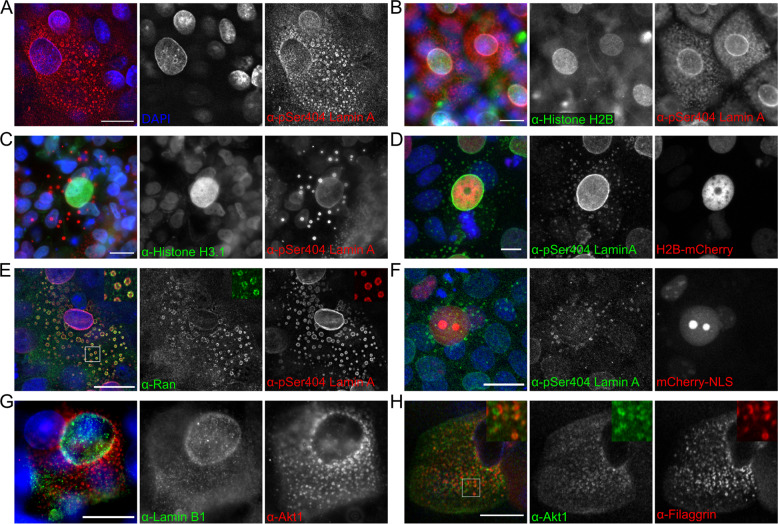
Lamina protein dispersal to the cytoplasm is pSer404 Lamin A/C specific and the cytoplasmic structures do not contain histone proteins, NLS-targeted protein or Lamin B1. **A** pSer404 Lamin A/C in suprabasal REKs co-stained with DAPI (DNA marker). Confocal z-section. pSer404 Lamin A/C in suprabasal REKs co-stained for Histone H2B (**B**) and Histone H3 (**C**). Epifluorescence images. **D** Histone H2B-mCherry positive post-confluent REKs stained for pSer404 Lamin A/C. Maximum projection of confocal z-sections, scale bar = 10 µm. **E** pSer404 Lamin A/C in suprabasal REKs co-stained for Ran GTPase. Confocal z- section, boxed area is enlarged. **F** NLS-mCherry expressing REK co-stained with pSer404 Lamin A/C in suprabasal REKs. Maximum projection of confocal z-sections. **G** Lamin B1 in suprabasal REKs co-stained with Akt1. Epifluorescence image. **H** Post-confluent REK cultures co-stained for Akt1 and filaggrin. Scale bars = 20 µm.

The following commercially available antibodies were used: Actin (Sigma-Aldrich, A2066), β-Actin (Millipore, MAB1501R), Akt (Cell Signaling Technology, 4691), Akt1 (Cell Signaling Technology, 2967), Arp3 (Santa Cruz Biotechnology, sc-48344), COPS4 (Invitrogen, PA5- 57863), FAK1 (Abgent, AP7715a), FLAG (Sigma-Aldrich, F3165), GAPDH Millipore Sigma MAB374, GFP (Santa Cruz Biotechnology, sc-9996), Histone H2B (Santa Cruz Biotechnology, sc-515808), Histone H3.1 (Santa Cruz Biotechnology, sc-517576), HspB1 (Abcam, ab12351), Jup (Santa Cruz Biotechnology, SC-514115), K10 (BioLegend, 905401), Lamin A (Santa Cruz Biotechnology; sc-376248, sc-398927, sc-518013), Lamin B1 (Abcam, ab16048), Loricrin (BioLegend, 905101), Myh9 (GeneTex, GTX113236), phospho-(Ser/Thr) Akt substrate (Cell Signaling Technology, 9611), Plectin (Santa Cruz Biotechnology, sc-33649), Ran (Santa Cruz Biotechnology, sc-271376) and STAMBP (Biorbyt, ORB341234). An antibody to pSer404 of Lamin A was raised in rabbit to phosphopeptide CGRASp-SHSSQTQGGG by Mimotopes (UK) Ltd, and another was a gift from Sandra Marmiroli (Figs. 1D, 2D and F, 7A). Alexa Fluor 568 Phalloidin was from Thermo Fisher Scientific. EGFP-Lamin A was a gift from Pekka Taimen. Lamin A-mEmerald (mEmerald-LaminA-N-18) and NLS-mCherry (mCherry-Nucleus-7) were gifts from Michael Davidson (Addgene plasmids #54139 and #55110, unpublished). Histone H2B-mCherry was a gift from Robert Benezra (Addgene plasmid #20972, [15]). FLAG-tagged S404A, S404D and WT Lamin A constructs were a gift from Sandra Marmiroli. shRNA constructs were obtained from QIAGEN (SureSilencing Akt1 shRNA plasmids) or Origene (HuSH Myh9 shRNA constructs).

### Cell culture

REKs were cultured in DMEM supplemented with 10% FBS, 1% penicillin/streptomycin at 37 °C and 5% CO_2_ and tested regularly to ensure cultures are free from *Mycoplasma* infection. Normal human epidermal keratinocytes were cultured in Epilife medium (Sigma Aldrich) and keratinocytes were differentiated by treatment with 1.2 mM calcium chloride for 4 days. For transfection, cells were transfected with Lipofectamine 2000 (Invitrogen) or jetOptimus (Polyplus transfection) according to manufacturers’ guidelines one day after seeding. After transfection with Akt1 (Qiagen) [16] or Myh9 shRNA plasmids (HuSH constructs, Insight Biotechnology), knockdown REKs were selected for by addition of G418 (400 µg/ml) or puromycin (1.25 µg/ml), respectively, to the medium for 10 days. Upon confluency REKs were fed every day and cultures fixed 2–4 days post confluency to analyse differentiation. Organotypic models were cultured and processed as described [10]. Treatments were performed upon confluency for 24 h: 10 nM Rapamycin (Cell Signaling Technology, 9904S), 50 µM Blebbistatin (Millipore, 203389), 1 µM Latrunculin B (Calbiochem, 428020), 50 µM CK666 (Abcam, ab141231) and 20 µM Q-VD-OPh.

### Western blotting

Cells were lysed in total lysis buffer (20% β-mercaptoethanol, 5% sodium dodecyl sulphate (SDS), 10 mM Tris pH 7) and boiled for 10 min at 95 °C. Protein lysates were separated on 4-20% SDS-polyacrylamide gels (Bio-Rad) and transferred to nitrocellulose membrane before blocking and with 5% milk (Marvel) or bovine serum albumin (Sigma) w/v in PBS-T (0.1% Tween-20 in PBS). The blocked membrane was probed with primary and HRP-linked secondary antibodies (Dako) diluted in the blocking buffer. Luminol (Santa Cruz Biotechnology) was used to detect protein bands and results were analysed with Fiji [17].

### Co-immunoprecipitation

Cells were lysed in RIPA buffer (50 mM Tris-HCl pH 7.4, 150 mM NaCl, 1% Triton X-100, 0.1% sodium deoxycholate, 0.5% SDS) and immunoprecipitation carried out using the Dynabeads Protein G Immunoprecipitation Kit (Thermo Fisher Scientific) according to the manufacturer’s instructions. Eluted proteins and controls were analysed by western blotting or Coomassie Blue staining (Fisher).

### Immunocytochemistry

REK monolayer cultures were fixed in 4% PFA 0.2% triton X-100 or −20 °C methanol. Blocking was performed with 0.4% fish skin gelatin in PBS with 0.2% Triton X-100 and primary and Alexa fluor-conjugated secondary antibodies were diluted in blocking solution. Samples were counterstained with DAPI, mounted with Prolong Gold (Life Technologies) and imaged on Leica DM5000B epifluorescence (Leica Microsystems, Milton Keynes, UK) and Zeiss LSM880 Airyscan (Zeiss, Cambridge, UK) laser confocal microscopes. Analysis was performed in Fiji; pSer404 Lamin A dispersing cells were counted using the ImageJ Cell Counter plugin, Lamin B1 shrunken nuclei were thresholded and then counted using the Analyze Particles plugin.

### Fluorescent immunohistochemistry

Organotypic culture sections, wild-type neonatal C57BL6 murine sections, and human skin sections (5 µm) were dewaxed and antigens retrieved in boiling 0.01 M sodium citrate pH 7 for 7 min. 0.4% fish skin gelatin 0.2% Triton X-100 in PBS blocking buffer was also used for primary and secondary antibody dilutions. Samples were mounted with Prolong Gold with DAPI (Life Technologies) and imaged on Leica DM5000B epifluorescence and Zeiss LSM880 Airyscan confocal microscopes.

### Live imaging

REKs transfected with fluorescently tagged proteins were seeded in CELLview™ Cell Culture Dishes and allowed to reach confluency and then cultured for a further 3-4 days before imaging. Confocal images were taken every 15/20 min on a Zeiss LSM880 Airyscan confocal microscope. Images were processed and analysed using Fiji.

### Flow cytometry and cell sorting

Post-confluent REK cultures were dissociated in trypsin, resuspended in PBS with Hoechst 33342 and incubated for 45 min at 37 °C. Flow cytometry was performed on a FACS Canto II (BD Biosciences, San Jose, USA) and analysed with FlowJo (BD). Cells were sorted, without Hoechst incubation, on a FACSAria IIIu Cell Sorter (BD Biosciences).

### Mass spectrometry

Collodial coomassie dye stained acrylamide gel bands were destained and digested using the method as reported [18]. The extracted peptides were further cleaned using C18 + carbon top tips (Glygen corporation, TT2MC18.96) and eluted with 70% acetonitrile (ACN) with 0.1% formic acid.

Dried peptides were dissolved in 0.1% TFA and analysed by nanoflow ultimate 3000 RSL nano instrument was coupled on-line to a Q Exactive plus mass spectrometer (Thermo Fisher Scientific, Waltham, USA). Gradient elution was from 3% to 35% buffer B in 120 min at a flow rate 250nL/min with buffer A being used to balance the mobile phase (buffer A was 0.1% formic acid in water and B was 0.1% formic acid in ACN). The mass spectrometer was controlled by Xcalibur software (version 4.0) and operated in the positive mode. The spray voltage was 1.95 kV and the capillary temperature was set to 255 °C. The Q-Exactive plus was operated in data dependent mode with one survey MS scan followed by 15 MS/MS scans. The full scans were acquired in the mass analyser at 375–1500 m/z with the resolution of 70,000, and the MS/MS scans were obtained with a resolution of 17,500.

MS raw files were converted into Mascot Generic Format using Mascot Distiller (version 2.5.1) and searched against the SwissProt database restricted to Rat entries using the Mascot search daemon (version 2.5.0) with a FDR of ~1% and restricted to the human entries. Allowed mass windows were 10 ppm and 25 mmu for parent and fragment mass to charge values, respectively. Variable modifications included in searches were oxidation of methionine, pyro-glu (N-term) and phosphorylation of serine, threonine and tyrosine. The mascot result (DAT) files were extracted into excel files for further normalisation and statistical analysis.

## Results

### Akt1-dependent dispersal of phosphorylated lamina proteins in keratinocyte differentiation

Akt1 is required for Lamin A/C phosphorylation and Akt1 knockdown prevents Lamin A/C degradation and nuclear removal, suggesting Lamin A/C phosphorylation is important for nuclear lamina breakdown in keratinocyte nuclear removal [10]. Phosphorylated serine 404 (pSer404) Lamin A/C disperses to intracellular structures in granular layer cells in human and murine skin sections and REK organotypic cultures [10], (Supplementary Fig. 1A). We determined that pSer404 Lamin A/C dispersal to cytoplasmic structures also occurred in suprabasal, differentiating REKs in monolayer culture, (Fig. 1A, B) and calcium switched human keratinocytes, (Fig. 1C), although these structures were less developed. Akt1 co-localised with pSer404 Lamin A/C at the nucleus and at dispersed structures throughout the cytoplasm, (Fig. 1D). Lamin A/C dispersal was reduced in Akt1 knockdown REK organotypic cultures [10], suprabasal Akt1 knockdown REKs had increased nuclear size in post-confluent cultures compared to controls [10], (Fig. 1E–H), and suprabasal differentiating cells are larger and less granular based on flow cytometry side scatter (SSC) measurements, (Fig. 1G–L). Conversely, rapamycin treatment, which increases Akt1 activity [19], increased the number of cells with Lamin A/C dispersal, (Fig. 1M, N). Akt1 activity is therefore required for Lamin A/C dispersal and nuclear degradation. Overexpression of myristoylated Akt1 (Myr-Akt1) did not increase the number of cells with Lamin A/C dispersal, but accumulated at the nucleus and increased pSer404 Lamin A/C intensity, suggesting a role for Akt1 in initiation of Lamin A/C dispersal before a separate dispersal step, (Supplementary Fig. 1B–D)

Overexpression of ‘non-AKT-phosphorylatable’ S404A Lamin A decreased Lamin A/C dispersal, (Fig. 1O, P), and reduced expression of the keratinocyte differentiation marker loricrin, (Supplementary Fig. 1E, F). This suggested that Akt1-dependent phosphorylation of Lamin A/C also affected differentiation. Overexpression of ‘phosphomimetic’ S404D Lamin A did not increase Lamin A/C dispersal, nor did it increase Loricrin staining or alter nuclear size, (Supplementary Fig. 1G–I). This construct did have a lower transfection efficiency, (Supplementary Fig. 1J), suggesting an intolerance to the expression of the phosphomimetic construct, or alternatively, the majority of Lamin A/C is phosphorylated, and so the system is not affected by the phosphomimetic.

### Lamina dispersal is specific to Lamin A/C

Dispersed pSer404 Lamin A/C structures did not contain any identifiable nuclear content; they did not consistently stain with the DNA intercalating dye DAPI and did not co-localise with Histone proteins, either endogenous Histone H3 or H2B or an overexpressed Histone H2B-mCherry, (Fig. 2A–d). Ran, a nuclear transport protein, dispersed to pSer404 Lamin A/C cytoplasmic structures, (Fig. 2E), indicating their origin at the nucleus. However, the structures did not contain mCherry targeted to the nucleus with a nuclear localisation sequence (NLS-mCherry), (Fig. 2F); indicating these structures did not contain functional nuclear pores. Additionally, Lamin B1 did not disperse to cytoplasmic Akt1-containing structures, (Fig. 2G); suggesting Lamin A/C but not Lamin B1 is involved in this process.

Dispersal of Akt1 and pSer404 Lamin A/C was similar to the previously identified cytoplasmic distribution of filaggrin in differentiating keratinocytes [20] and Akt1 showed some co-localisation with filaggrin in suprabasal keratinocytes, (Fig. 2H).

### Akt1-associated proteins form a ‘degradosome’ complex in differentiating REKs

**Fig. 3:**
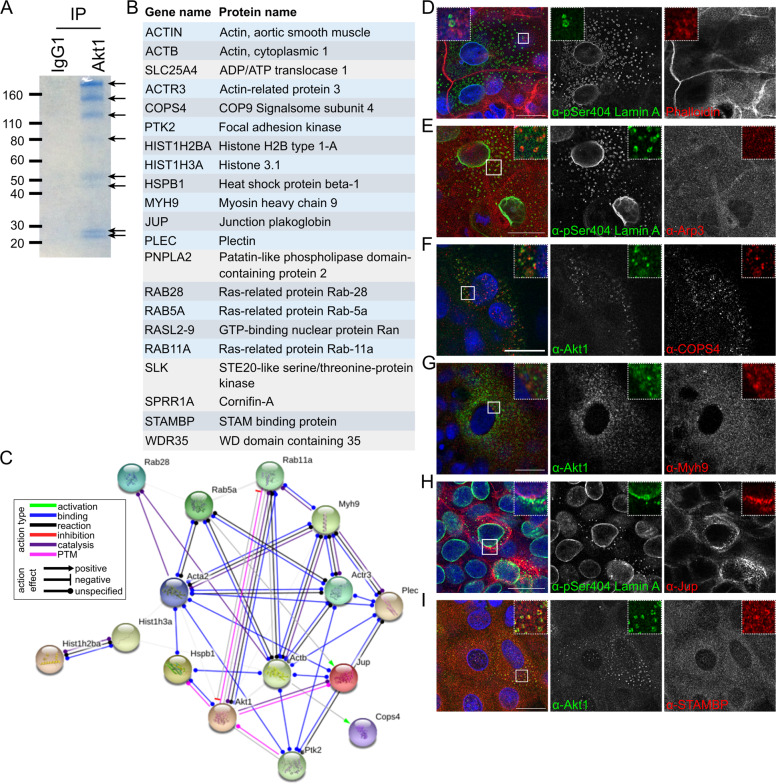
Identification of Akt1 interactors and co-staining with Akt1 and pSer404 Lamin A in differentiating keratinocytes. **A** Coomassie staining of IgG1 and Akt1 immunoprecipitate (IP). **B** List of Akt1 interactors identified by LC-MS/MS of Akt1 co-immunoprecipitate. **C** STRING analysis of interactors in **A**. PTM = posttranslational modification. Suprabasal differentiating REKs with Akt1 or pSer404 Lamin A/C co-stained for actin (phalloidin) (**D**), Arp3 (**E**), COPS4 (**F**), Myh9 (**G**), Jup (**H**), STAMBP (**I**). Confocal z-sections, scale bars = 20 µm.

**Fig. 4:**
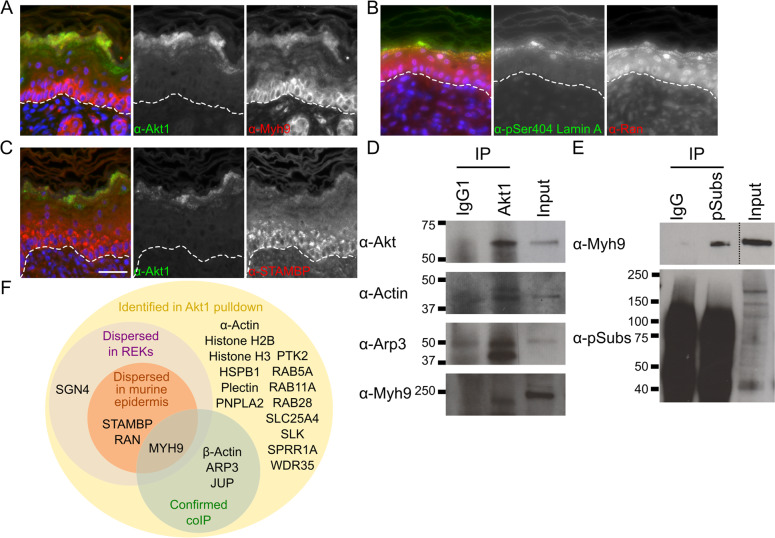
Akt1 interactors in murine epidermis and in Akt1 immunoprecipitate. Neonatal wild-type murine epidermis stained for Akt1 or pSer404 Lamin A and co-stained for Myh9 (**A**), Ran (**B**), STAMBP (**C**). **D** IgG1 and Akt1 immunoprecipitate immunoblotted for total Akt, actin, Arp3 and Myh9. **E** IgG and phospho-(Ser/Thr) Akt substrate (pSubs) IPs immunoblotted for Myh9 and pSubs. **F** Summary of Akt1 interactors identified by mass spectrometry in an Akt1 co-immunoprecipitate that were also detected to be dispersed with Akt1 and pSer404 Lamin A/C in REKs, murine epidermal sections, and confirmed by western blotting of Akt1 co-immunoprecipitate.

AKT family kinases have a large number of downstream target proteins involved in a wide range of cellular processes that may be tissue, cell and cellular location specific [21], suggesting the potential of novel Akt1-interacting proteins being involved in lamin dispersal and nuclear shrinkage. To explore this we performed immunoprecipitation with an Akt1-specific antibody, followed by mass spectrometric analysis of eight major SDS-PAGE gel bands. Excluding mitochondrial and keratin proteins, we identified 21 potential Akt1-interacting partners in REKs (Fig. 3A, B), including the previously identified Akt1 target, HspB1 [9]. STRING analysis of these candidates identified several previously identified interactions or links between these proteins, (Fig. 3C). Co-staining with Akt1 or pSer404 Lamin A antibodies demonstrated co-localisation or adjacent expression on cytoplasmic structures for filamentous actin (phalloidin), Arp3, Cops4, Myh9, Ran, Jup and Stambp, (Figs. 2E and 3D–I). Staining of mouse epidermis indicated co-expression of dispersed Akt1 or pSer404 Lamin A staining with Myh9, Ran and Stambp in suprabasal keratinocyte layers, (Fig. 4A–C). Immunoprecipitation confirmed β-actin, Arp3, and Myh9 interacted with Akt1 (Fig. 4D). The difference in apparent molecular weight for immunoprecipitated Myh9 may be due to phosphorylation, potentially Akt1-dependent phosphorylation, as Myh9 was pulled down by an antibody directed to Akt phosphorylation sites, (Fig. 4E). These results suggest functionally related Akt1 interactors suggestive of a ‘degradosome’ complex including actin and the actin-binding proteins, Myh9 and Arp3, associating with Akt1 and with cytoplasmic pSer404 Lamin A in differentiating REKs, (Fig. 4F). Myh9 is a component of myosin IIa, a molecular motor that can translocate along actin filaments, and Arp3 is a component of the Arp2/3 complex required for actin filament nucleation [22, 23]. This suggests a dependence on the actin cytoskeleton and actin-binding proteins for Akt1 function in epidermal terminal differentiation.

### Akt1-associated actin remodelling is required for nuclear degradation

**Fig. 5:**
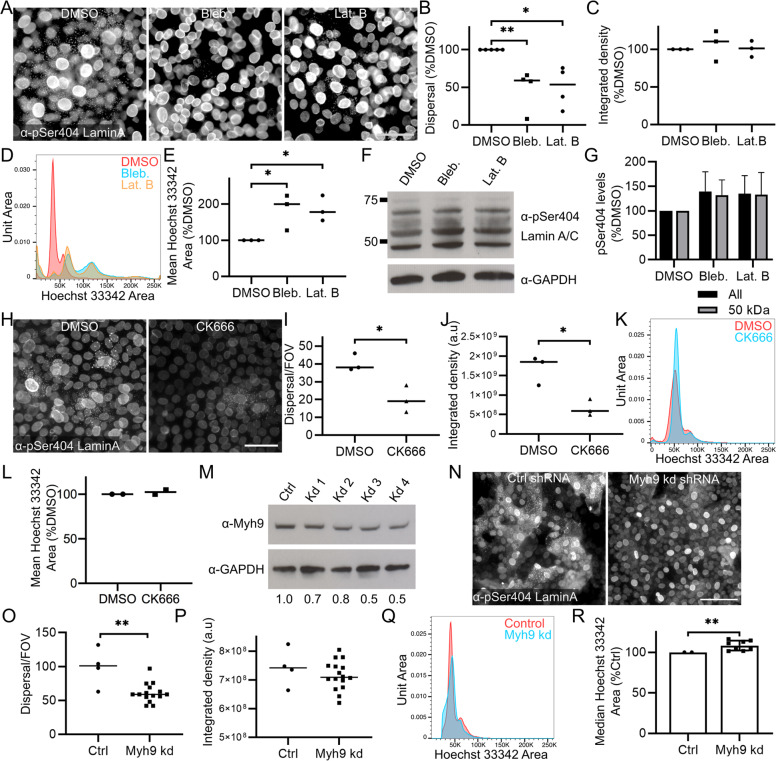
Disruption of cytoskeletal Akt1 target protein function affects nuclear lamina dispersal and nuclear size. **A** DMSO, blebbistatin and latrunculin B treated REKs stained for pSer404 Lamin A/C. Epifluorescence images, scale bar = 50 µm. **B** Number of cells with pSer404 Lamin A/C dispersal in DMSO, blebbistatin and latrunculin B treated REKs. % of DMSO control, >4 independent experiments, >3 FOV per experiment, one-way ANOVA *p ≤ 0.05, **p ≤ 0.01. **C** pSer404 Lamin A/C intensity in DMSO, blebbistatin and latrunculin B treated REKs. % of DMSO control, 3 independent experiments, >3 FOV per experiment, one-way ANOVA all comparisons non-significant. **D** Representative Hoechst 33342 Area of DMSO, blebbistatin and latrunculin B treated REKs. **E** Mean Hoechst 33342 Area of DMSO, blebbistatin and latrunculin B treated REKs. % of DMSO control, 3 independent experiments, one-way ANOVA, **p ≤ 0.01, ***p ≤ 0.001. **F** Representative pSer404 Lamin A immunoblots of DMSO, blebbistatin and latrunculin B treated REKs. **G** pSer404 Lamin A level of all fragments and the ~50 kDa fragment. % of DMSO, 2 independent experiments, all comparisons ns. **H** DMSO or CK666 treated REKs stained for pSer404 Lamin A/C. Epifluorescence images, scale bar = 50 µm. **I** Number of cells with pSer404 Lamin A/C dispersal in DMSO and CK666 treated REKs. 3 FOV, unpaired *t*-test *p ≤ 0.05. **J** pSer404 Lamin A/C staining intensity in DMSO and CK666 treated REKs. 3 FOV, unpaired *t*-test *p ≤ 0.05. **K** Representative Hoechst 33342 area of DMSO and CK666 treated REKs. **L** Median Hoechst 33342 area of DMSO and CK666 treated REKs. % of DMSO control, 2 independent experiments, non-significant. **M** Myh9 expression in Ctrl and Myh9 shRNA knockdown post-confluent REKs. Numbers are relative to GAPDH expression and normalised to Ctrl. **N** Ctrl and Myh9 shRNA knockdown post-confluent REKs stained for pSer404 Lamin A/C. Epifluorescence images, scale bar = 50 µm. **O** Number of cells with pSer404 Lamin A/C dispersal in Ctrl and Myh9 shRNA knockdown REKs. 4 independent shRNA knockdown cell lines, >3 FOV per line, Mann–Whitney test, **p ≤ 0.01. Representative of two independent experiments. **P** pSer404 Lamin A/C staining intensity in control and Myh9 shRNA knockdown cells. 4 independent shRNA knockdown cell lines, >3 FOV per line, Mann–Whitney test non-significant. Representative of two independent experiments. **Q** Representative Hoechst 33342 area of Ctrl and Myh9 shRNA knockdown REKs. **R** Median Hoechst 33342 area in Ctrl and Myh9 shRNA knockdown REKs. 2 independent experiments, 4 independent shRNA knockdown cell lines, error bars = SD, Wilcoxon test, **p ≤ 0.01.

To test the requirement for actin and myosin II motors in nuclear degradation, we chemically disrupted their function; using latrunculin B to block actin polymerisation and blebbistatin to inhibit myosin II activity in post-confluent REKs. Latrunculin B and blebbistatin treatment inhibited Lamin A/C dispersal to the cytoplasm, (Fig. 5A, B), and increased nuclear size in large granular differentiating REKs, (Fig. 5D–E and Supplementary Fig. 2A, B). These treatments did not affect levels of phosphorylated full length, and cleaved, Lamin A/C in post-confluent REK cultures, (Fig. 5C, F, G). Treatment with CK666, an Arp2/3 specific inhibitor decreased phosphorylated Lamin A/C and dispersal at 50 µM, (Fig. 5H–J) but had no significant effect on nuclear size, (Fig. 5K, L and Supplementary Fig. 2C).

Myh9 (myosin IIa) shRNA knockdown (Fig. 5M) also decreased phosphorylated Lamin A/C dispersal, (Fig. 5N–P) and increased nuclear size, (Fig. 5Q–R). This suggested that the activity of myosin II and the presence of the actin cytoskeleton is required for the formation and/or distribution of cytoplasmic Lamin A/C structures after Akt1-dependent phosphorylation of Lamin A/C.

### Fluorescently tagged nuclear proteins illustrate nuclear shrinkage in REKs

**Fig. 6:**
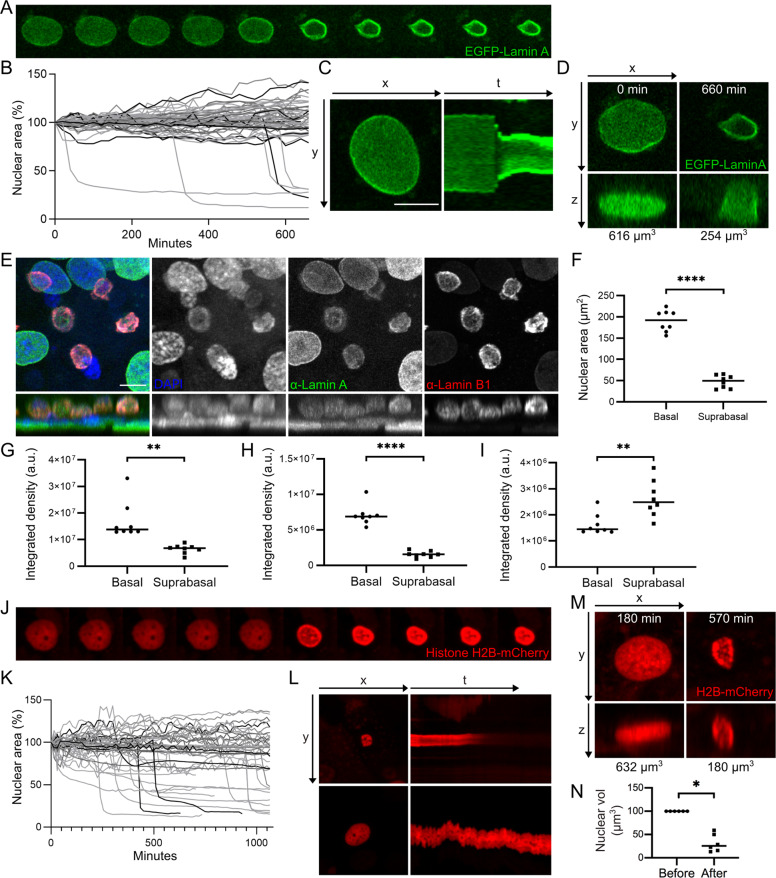
Nuclear dynamics in differentiating REKs. **A** Images every 20 min of EGFP-Lamin A positive post-confluent REKs. **B** Cross-sectional area EGFP-Lamin A positive nuclei over time. **C** Kymographs (yt projection) of a EGFP-Lamin A positive nucleus over time. **D** Xz projections of a EGFP-Lamin A positive nucleus. Labelled with nuclear volume. **E** Xy and xz projections of confocal z-sections of post-confluent REKs stained for Lamin A and Lamin B1. Scale bar = 10 µm. **F** Cross-sectional area of basal and suprabasal nuclei in post-confluent REKs. Two independent experiments, Welch’s *t* test, ****p ≤ 0.0001. Intensity of DAPI (**G**), Lamin A (**H**) and Lamin B1 (**I**) staining in basal and suprabasal nuclei in post-confluent REK cultures. Welch’s *t* test, **p ≤ 0.01, ****p ≤ 0.0001. Representative of two independent experiments. **J** Images every 15 min of Histone H2B-mCherry positive post-confluent REKs. **K** Cross-sectional area over time in 45 Histone H2B-mCherry positive nuclei greater than 80 µm^2^. **L** Kymograph (yt projection) of Histone H2B-mCherry over time; shrunken nucleus (top panels) and a nucleus that does not shrink (bottom panels). **M** Xz projections of Histone H2B-mCherry positive nuclei labelled with nuclear volume. **N** Histone H2B-mCherry positive nuclear volume before and after shrinkage. Wilcoxon test, *p ≤ 0.05.

To understand how Lamin A/C dispersal occurs in suprabasal differentiating REKs we expressed fluorescently tagged nuclear proteins and imaged at least three days post-confluency to follow changes in nuclear morphology in real time. The majority of EGFP-Lamin A positive REKs did not alter in nuclear size/maximum cross-sectional area over 11 h, (Movie 1). However, 10% of nuclei rapidly decreased in size, within 40 min, (Movie 2 and Fig. 6A–C). There is no further decrease in size after this, (Fig. 6B, C), and this is a decrease in nuclear volume not just due to rotation of the nucleus, (Fig. 6D). There was no change in signal intensity in EGFP-Lamin A expressing nuclei that had shrunk (Fig. 6C), although, DAPI and Lamin A/C staining intensity decreased in suprabasal shrunken nuclei compared to basal ‘non-shrunken nuclei’ in fixed post-confluent REKs, (Fig. 6E–H). Conversely, apparent Lamin B1 staining intensity increased in shrunken nuclei, (Fig. 6I).

In post-confluent H2B-mCherry expressing REKs approximately one quarter of labelled nuclei rapidly decreased in surface area, (Fig. 6J, K and Supplementary Fig. 3A–C), with an average decrease in nuclear volume of 68.5%, (Fig. 6M, N). mCherry signal intensity decreased over time in shrunken nuclei, compared to ‘non-shrunken’ nuclei, until the signal was lost in 41.6% of shrunken nuclei, (Fig. 6L and Supplementary Fig. 3C). Similarly to nuclei expressing EFGP-Lamin A, there was no decrease in mCherry or EGFP signal over 10 h in shrunken nuclei of REKs co-expressing Histone H2B-mCherry and EGFP-Lamin A (Supplementary Fig. 3D–F). This indicated that nuclear shrinkage occurs before nuclear content degradation, and continued expression of Lamin A/C prevented nuclear degradation after shrinkage.

### Phosphorylated Lamin A/C is cleaved prior to cytoplasmic dispersal

**Fig. 7:**
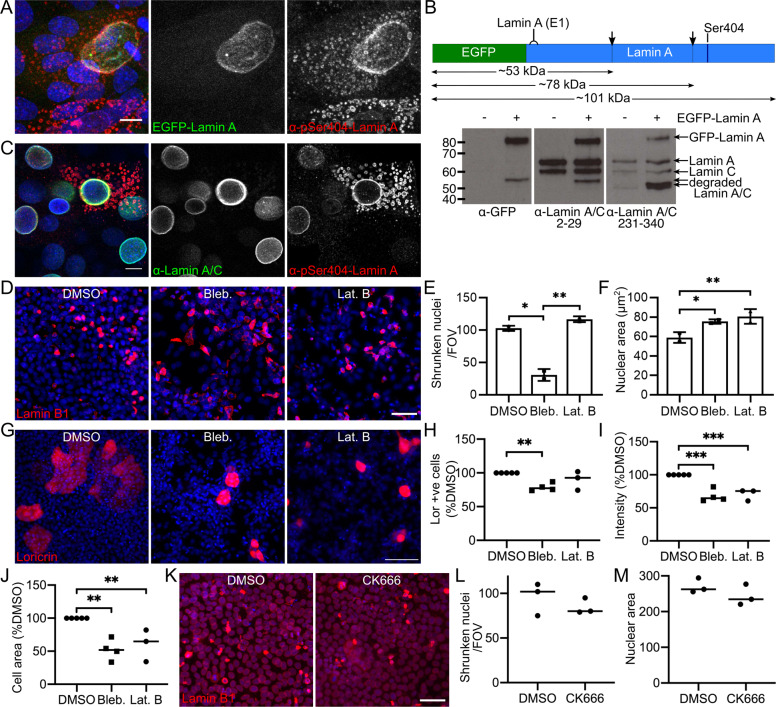
Lamin A cleavage in nuclear degradation and cytoskeletal protein inhibitors prevent nuclear shrinkage. **A** EGFP-Lamin A post-confluent REKs stained for pSer404 Lamin A. Maximum projection of confocal z-sections, scale bar = 10 µm. **B** Diagram of Lamin A primary protein structure, highlighting predicted cleavage sites (arrows) and N-terminal antigen for antibody (E1) and immunoblots of untransfected and EGFP-Lamin A transfected post-confluent REK lysates with antibodies directed to GFP and two Lamin A/C regions. **C** Post-confluent REKs stained for pSer404 Lamin A and the N-terminus of Lamin A/C. Single confocal plane, scale bar = 10 µm. **D** DMSO, blebbistatin and latrunculin B treated REKs stained for Lamin B1. Scale bar = 50 µm. Number per FOV (**E**) and area (**F**) of shrunken Lamin B1 expressing nuclei in DMSO, blebbistatin and latrunculin B treated REKs. 2 experiments, 3 FOV/experiment, error bars = SD, two-way ANOVA, *p ≤ 0.05, **p ≤ 0.01. **G** DMSO, blebbistatin and latrunculin B treated REKs stained for loricrin. Scale bar = 100 µm. **H** Number of loricrin positive cells per FOV in DMSO, blebbistatin and latrunculin B treated REKs. >3 experiments, 3 FOV/experiment, one-way ANOVA, **p ≤ 0.01. **I** Loricrin staining intensity in DMSO, blebbistatin and latrunculin B treated REKs. % of DMSO control, >3 experiments, 3 FOV/experiment, one-way ANOVA, ***p ≤ 0.001. **J** Cell area of loricrin positive cells in DMSO, blebbistatin and latrunculin B treated REKs. >3 experiments, 3 FOV/experiment, one-way ANOVA, **p ≤ 0.01. **K** DMSO and CK666 treated REKs stained for Lamin B1. Scale bars = 50 µm. **L** Number of shrunken Lamin B1 expressing nuclei per FOV in DMSO and CK666 treatments. 3 FOV, all comparisons non-significant. **M** Nuclear area of shrunken Lamin B1 expressing nuclei in DMSO and CK666 treatments. 3 FOV, all comparisons non-significant.

Interestingly, EGFP-Lamin A did not disperse to cytoplasmic structures in live imaging experiments and EGFP-Lamin A did not co-localise with dispersed pSer404 Lamin A, (Fig. 7A), suggesting that cleavage of Lamin A may occur before dispersal to the cytoplasm. Two cysteine protease cleavage sites have been predicted for Lamin A/C: Asp230 and Asp446, with cleavage during apoptosis at Asp230 [24, 25]. Western blots of post-confluent EGFP-Lamin A expressing REKs (N-terminal EGFP tag) and in Lamin A-mEmerald expressing REKs (C-terminal Emerald tag) demonstrated Lamin A fragments of approximately 55 and 80 kDa, respectively, which would be consistent with cleavage of Lamin A around Asp230, (Fig. 7B and Supplementary Fig. 4A–C). An antibody targeted to the Lamin A/C N-terminus did not stain cytoplasmic pSer404 Lamin A structures, (Fig. 7C). This indicates only the C-terminal Lamin A/C degradation product, containing the Ser404 phosphorylation site, is targeted to cytoplasmic structures.

Nuclei expressing C-terminally tagged Lamin A/C, Lamin A-mEmerald, underwent nuclear shrinkage with the same kinetics as EGFP-Lamin A expressing REKs, (Supplementary Fig. 4D–G), but Lamin A-mEmerald also did not disperse, (Supplementary Fig. 4H). Lamin A/C contains a C-terminal CAAX box required for farnesylation before prelamin A/C cleavage, which is necessary for functional integration into the nuclear lamina [26–28]. Prevention of CAAX box modification and/or cleavage by presence of the C-terminal mEmerald tag may prevent correct Lamin A-mEmerald integration into the nuclear lamina. Consistent with this, nuclei expressing Lamin A-mEmerald had a disrupted nuclear lamina compared to EGFP-Lamin A expressing nuclei, (Supplementary Fig. 4I). Caspase inhibition did not affect pSer404 Lamin A dispersal in post-confluent REK cultures (Supplementary Fig. 4J, K).

### The actomyosin cytoskeleton is required for nuclear shrinkage and epidermal differentiation

We tested the dependence of nuclear shrinkage on the activity of Akt1-associated cytoskeletal proteins. Blebbistatin treatment reduced the number and increased the nuclear size of shrunken Lamin B1-positive nuclei, (Fig. 7D–F), indicating myosin II activity is required for Lamin A/C dispersal and nuclear shrinkage. Latrunculin B treatment also increased the size of nuclei that had high nuclear Lamin B1 expression, (Fig. 7D, F), but latrunculin B and CK666 treatment did not affect the number of cells with high Lamin B1 expression, (Fig. 7E, K–M). Both blebbistatin and latrunculin B treatments reduced loricrin expression and affected differentiated keratinocyte morphology, reducing the size of loricrin expressing keratinocytes, (Fig. 7G–J).

**Fig. 8:**
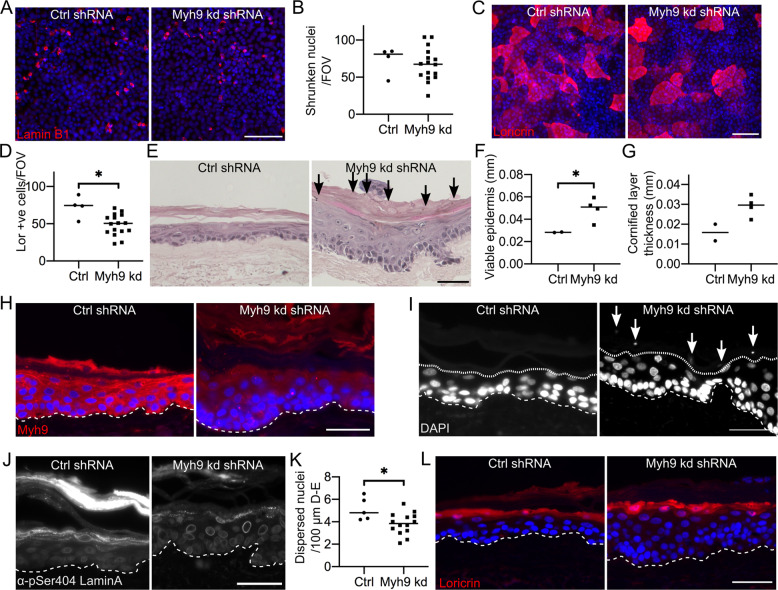
Myh9 knockdown disrupts nuclear degradation and differentiation. **A** Ctrl and Myh9 shRNA knockdown post-confluent REKs stained for Lamin B1. Scale bar = 100 µm. **B** Number of shrunken Lamin B1 expressing nuclei per FOV in Ctrl and Myh9 shRNA knockdown post-confluent REKs. 4 independent shRNA knockdown cell lines, >3 FOV per line, unpaired t-test, non-significant. **C** Ctrl and Myh9 shRNA knockdown post-confluent REKs stained for loricrin. Scale bar = 100 µm. **D** Number of loricrin positive cells per FOV in Ctrl and Myh9 shRNA knockdown post-confluent REKs. 4 independent shRNA knockdown cell lines, >3 FOV per line, Mann–Whitney test, *p ≤ 0.05. **E** Ctrl or Myh9 shRNA knockdown REK organotypics stained with hematoxylin and eosin (H&E). Arrows indicate retained nuclei, scale bar = 50 µm. Height of viable epidermis (**F**) and cornified layers (**G**) in H&E stained Ctrl and Myh9 shRNA knockdown organotypics. 2 organotypics per line, two independent Myh9 shRNA knockdown lines, >3 measurements per FOV, Welch’s *t* test, *p ≤ 0.05. **H** Ctrl and Myh9 shRNA knockdown organotypics stained for DAPI and Myh9. Scale bar = 50 µm. **I** Ctrl or Myh9 shRNA knockdown REK organotypics stained with DAPI. Dashed line marks dermal-epidermal junction, dotted line indicates junction of granular and cornified layers and arrows indicate retained nuclei, scale bar = 50 µm. **J** Ctrl or Myh9 shRNA knockdown REK organotypics stained for pSer404 Lamin A. Dashed line marks dermal-epidermal junction, scale bar = 50 µm. **K** Number of nuclei with phosphorylated Lamin A/C dispersal per 100 µm of the dermal-epidermal (**D** and **E**) junction in Ctrl or Myh9 shRNA knockdown organotypics. 2 independent shRNA knockdown cell lines, 2 organotypics per cell line, >3 FOV per line, Mann–Whitney test, *p ≤ 0.05. **L** Ctrl and Myh9 shRNA knockdown organotypics stained for loricrin. Scale bar = 50 µm.

Myh9 shRNA knockdown did not significantly affect nuclear shrinkage, (Fig. 8A, B), but did decrease loricrin expression, (Fig. 8C, D). Myh9 knockdown organotypic cultures had decreased Myh9 expression, (Fig. 8H), were hyperkeratotic and displayed small parakeratotic nuclear material, (Fig. 8E–G, I). Fewer cells underwent Lamin A/C dispersal, (Fig. 8J, K), but there was no detectable change in loricrin expression, (Fig. 8L). Defects in Lamin A/C dispersal and differentiation without defects in nuclear shrinkage may indicate that other mechanisms also affect nuclear shrinkage.

## Discussion

We present data suggesting that nuclear degradation in mammalian epidermal keratinocytes requires Akt1-dependent phosphorylation and cleavage of Lamin A/C before actin cytoskeleton and myosin II mediated cytoplasmic dispersal of pSer404 Lamin A/C. Lamina removal from the nucleus precedes nuclear shrinkage and then nuclear content degradation. Akt1 and its interactors act as part of a ‘degradosome’ complex required for two novel nuclear removal intermediate processes during keratinocyte nuclear destruction: Lamin A/C dispersal and rapid nuclear volume reduction.

Lamins A/C and B1/2 are important components of the nuclear lamina, where they regulate nuclear morphology [29–32] and control chromatin organisation with the lamin B receptor [33]. They interact with DNA through lamina-associated domains (LADs), which are enriched in transcriptionally repressed areas of the genome [34]. Various models of lamin association with LADs have been proposed, including direct tethering to chromatin domains [35] and a ‘meshwork caging model’ where the dense lamin network traps chromatin domains [36, 37]. Work in Lamin null mice demonstrated that loss of LADs altered chromatin organisation and affected gene expression in neighbouring ‘non-LAD’ genomic regions [38]. Interestingly, murine skin develops normally without Lamin B1 or B2 expression [39] and A and B-type lamins have been suggested to have different functions in chromatin organisation and gene expression regulation [40–42]. We demonstrated that Lamin A/C is specifically dispersed to cytoplasmic structures, which do not contain DNA, histone proteins, NLS-targeted protein or Lamin B1. The specific removal of Lamin A/C from the nuclear periphery may specifically alter keratinocyte gene expression to regulate subsequent stages in keratinocyte differentiation.

Our data suggests cleavage of Lamin A/C occurs prior to dispersal from the nucleus, and only the C-terminal fragment of Lamin A/C disperses to the cytoplasm. C-terminally tagged Lamin A-mEmerald was not dispersed, however, the C-terminal tag is likely to affect correct Lamin A/C processing at the C-terminal CAAX box [28]. Progerin, a C-terminal mutant form of Lamin A/C [43], retains modifications at the CAAX box and is not correctly cleaved, altering nuclear lamina structure and nuclear morphology [44, 45]. Therefore, the C-terminal mEmerald tag may prevent correct integration into the nuclear lamina; affect nuclear morphology, Lamin A/C phosphorylation and/or cleavage and dispersal.

We identified myosin IIA activity and the actin cytoskeleton as important for epidermal differentiation and Lamin A/C dispersal. Myosin II activity is required for nuclear repositioning in cell migration [46], constriction of the cytokinetic actomyosin contractile ring [47–49] and contraction of an actin network around the nucleus at nuclear envelope breakdown [50]. Arp2/3, required for branched actin filament nucleation, has previously been identified as required for epidermal differentiation [51, 52], regulation of nuclear actin [53] and correct formation of the actomyosin contractile ring in cell division [54]. Myosin IIA and Arp2/3 have also been identified as responsible for force generation in the secretion of vesicles in alveolar type II (ATII) cells [55] and myosin IIA and actin are required for exocytosis in murine exocrine cells [56]. Disrupting myosin II and Arp2/3 activity affected lamin dispersal, however, whether their activity is important for force generation for formation of cytoplasmic pSer404 Lamin A/C structures or for trafficking of these structures will be important to determine.

Dispersed Akt1 in the cytoplasm of differentiating keratinocytes also coincided with dispersed filaggrin, which may indicate filaggrin may also be a component of the Akt1-interacting complex or ‘degradosome’. Filaggrin processing is tightly regulated and required for healthy epidermal differentiation [57, 58]. We previously identified that upon decreased Akt1 activity, expression of Cathepsin H decreased, and Cathepsin H deficiency in heterozygous murine models impaired filaggrin processing, caused barrier defects and altered keratinocyte differentiation [16]. Additionally, overexpression of a filaggrin N-terminal construct in COS7 cells affected nuclear integrity, leading to punctate filaggrin and lamin immune-positive structures throughout the cytoplasm [59]. This also correlates with the actomyosin cytoskeletal dependence of Lamin A/C dispersal, as Akt1 influences filaggrin-actin interactions and is essential for filaggrin processing [9, 20]. The parallel structures and the changes in filaggrin localisation in Cathepsin H knockout mice may indicate that Akt1-dependent lamin degradation and control of filaggrin processing may be interlinked and would be an interesting avenue to pursue.

Our results suggest another nuclear degradation intermediate of a ‘shrunken nucleus’, which may be the major morphological alteration in nuclear removal. Dispersion of cleaved Lamin A/C without dispersal of nuclear contents prior to nuclear shrinkage suggests that degradation and removal of nuclear lamina proteins may be an initial step in the initiation of nuclear removal. REKs expressing GFP-tagged Lamin A undergo nuclear shrinkage, but do not undergo further degradation. REKs expressing Histone H2B-mCherry underwent nuclear shrinkage followed by a gradual decrease in signal intensity, suggesting degradation of nuclear contents. This suggests decreased expression of Lamin A/C at the nuclear lamina is required for nuclear content degradation. Lamin A/C and Lamin B1 form separate but interconnecting networks [60] and perform distinct functions at the nuclear lamina; Lamin A/C deficient nuclei have decreased nuclear stiffness and increased deformations whereas Lamin B1 deficient nuclei display normal nuclear mechanics [30]. Protein and lipid depleted nuclear matrices have been shown to reversibly shrink in response to bivalent cation concentration [61] or nuclease treatment [62, 63] and isolated rat nuclei shrink in response to elevated calcium levels [64]. Degradation of nuclear material occurs after Lamin A/C depletion of the nucleus, as DNase deficient murine epidermis retains DAPI but not Lamin A/C staining in corneocytes [11]. Therefore, removal of Lamin A/C may allow subsequent changes in nuclear morphology by altering the stiffness of the lamina, and/or by allowing access for molecules necessary for nuclear content degradation.

Overexpression of GFP-tagged Lamin A/C constructs inhibited nuclear content degradation and Akt1 or Myh9 knockdown organotypics contained parakeratotic nuclei, demonstrating impaired nuclear removal. This indicated that Lamin A/C cleavage and dispersal are required for complete nuclear removal. Disruption of myosin activity and the actin cytoskeleton, through blebbistatin, latrunculin B and CK666 treatment and Myh9 (myosin IIA) knockdown affected lamin dispersal and increased nuclear size in differentiating keratinocytes. However, only blebbistatin treatment affected the number of shrunken Lamin B1 expressing nuclei in our post-confluent REK cultures, suggesting that although removal of Lamin A/C from the nuclear periphery is essential for nuclear degradation and keratinocyte differentiation it is not required for nuclear shrinkage. Nuclear shrinkage may be independently controlled by myosin IIB and IIC, which have some partially redundant properties but distinct cellular localisations [65–69] to myosin IIA.

Targeted autophagy of the nucleus, nucleophagy, is required for keratinocyte nuclear removal; with localisation of LC3 and autophagosomes to indentations at the nuclear periphery of differentiating keratinocytes [12]. Additionally, impaired clearance of nuclear material by autophagosomes was identified in laminopathies, diseases with mutations in lamina proteins [13], suggesting that alterations to nuclear lamina structure are key for nuclear destruction. We suggest that dispersal of pSer404 Lamin A/C to cytoplasmic structures and nucleophagic processes at the nuclear periphery may act concurrently to complete nuclear removal.

Identification of the molecular mechanisms that initiate and are involved in nuclear removal would be important for understanding and treating parakeratotic skin diseases. However, this could also be of wider importance in other cell types to prevent cellular processes including proliferation and to activate cell death by programmed nuclear clearance. We hope that further delineation of the processes leading to nuclear removal in healthy epidermis will advance this aim.

## Supplementary information

Supplementary figure S1

Supplementary Figure S1 Legend

Supplementary figure S2

Supplementary Figure S2 Legend

Supplementary figure S3

Supplementary Figure S3 Legend

Supplementary figure S4

Supplementary Figure S4 Legend

Supplementary Movie 1

Supplementary Movie 2
