# Supplementary figures and images for: Akt1-associated actomyosin remodelling is required for nuclear lamina dispersal and nuclear shrinkage in epidermal terminal differentiation
Source: Cell Death Differ. 2021 Jan 18;28(6):1849–64. doi: 10.1038/s41418-020-00712-9 (PMC8184862; doi:10.1038/s41418-020-00712-9)

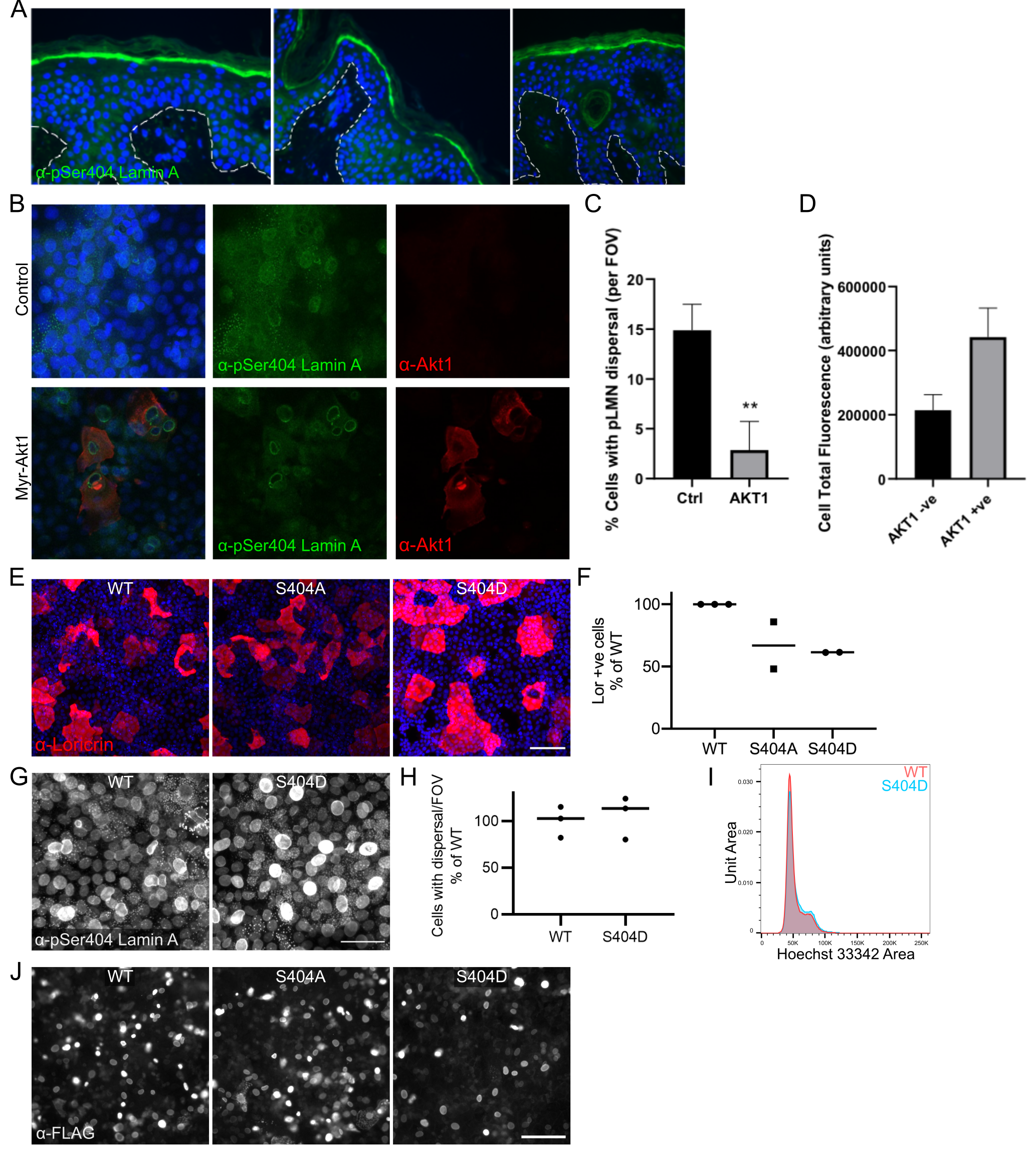

Supplement: Supplementary file 1 — Supplementary figure S1 [file 41418_2020_712_MOESM1_ESM.png]

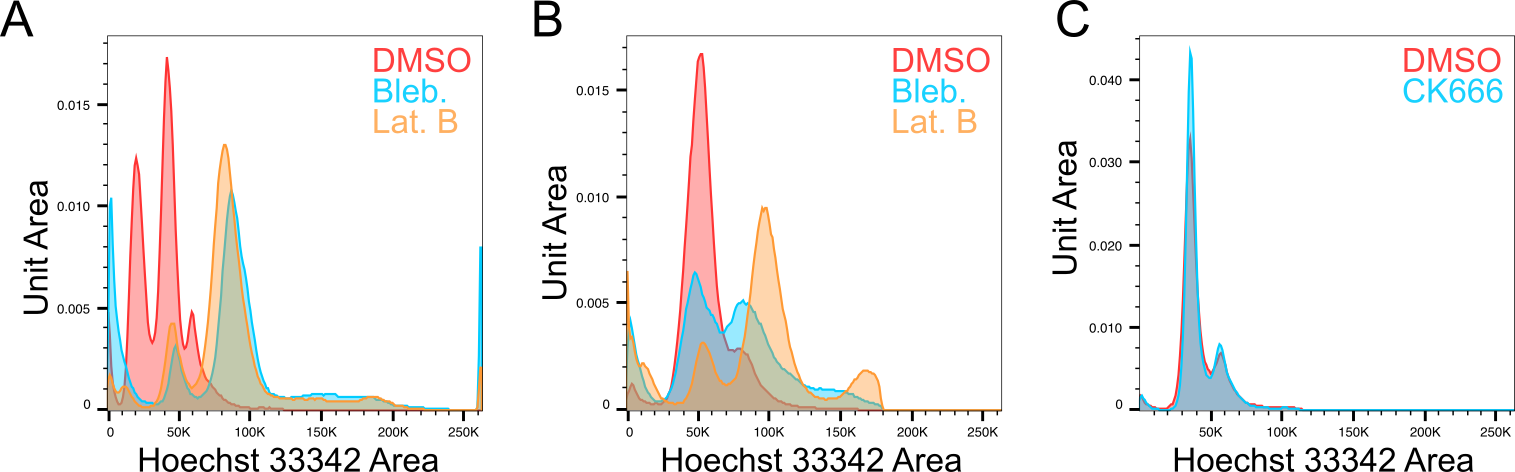

Supplement: Supplementary file 3 — Supplementary figure S2 [file 41418_2020_712_MOESM3_ESM.png]

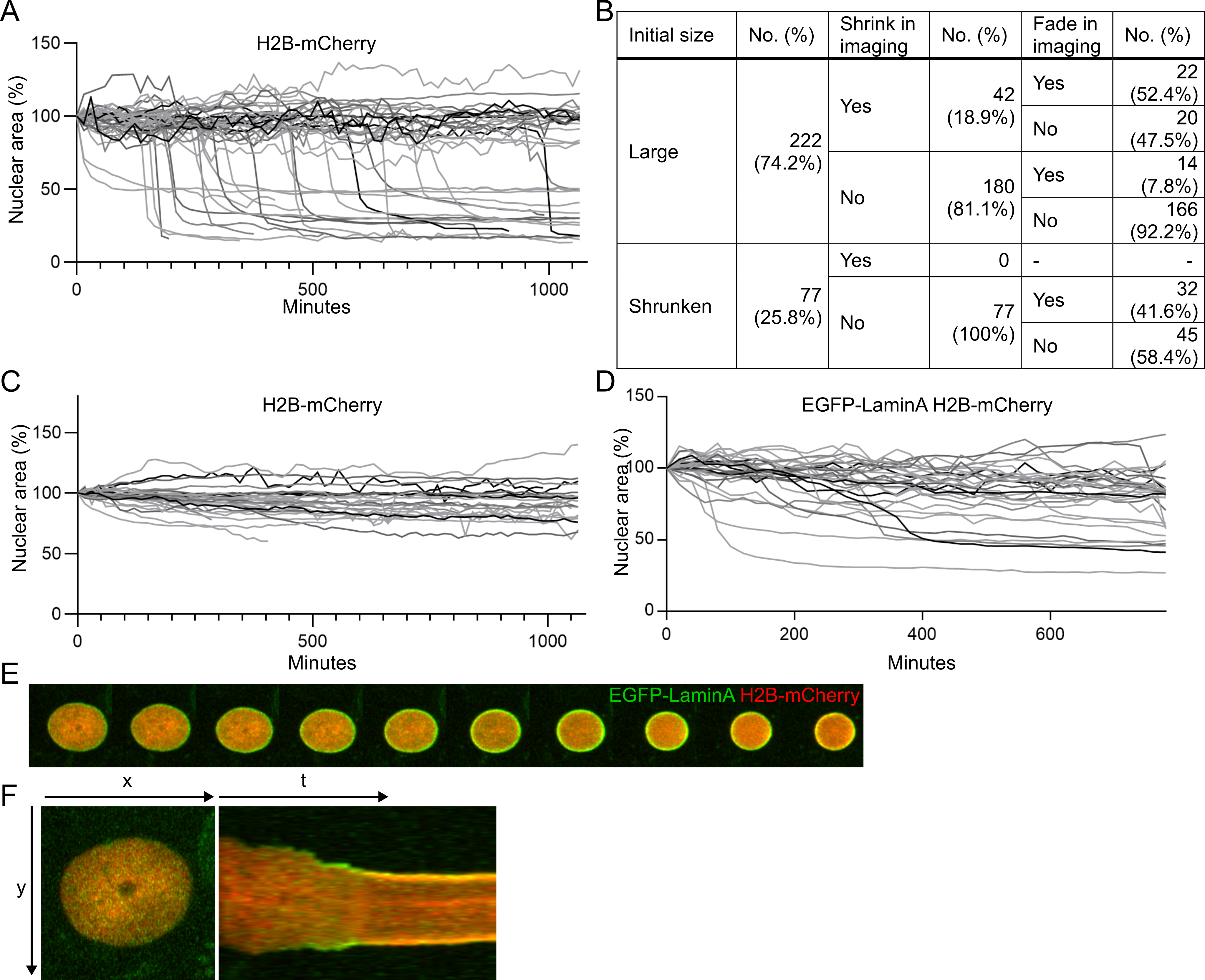

Supplement: Supplementary file 5 — Supplementary figure S3 [file 41418_2020_712_MOESM5_ESM.png]

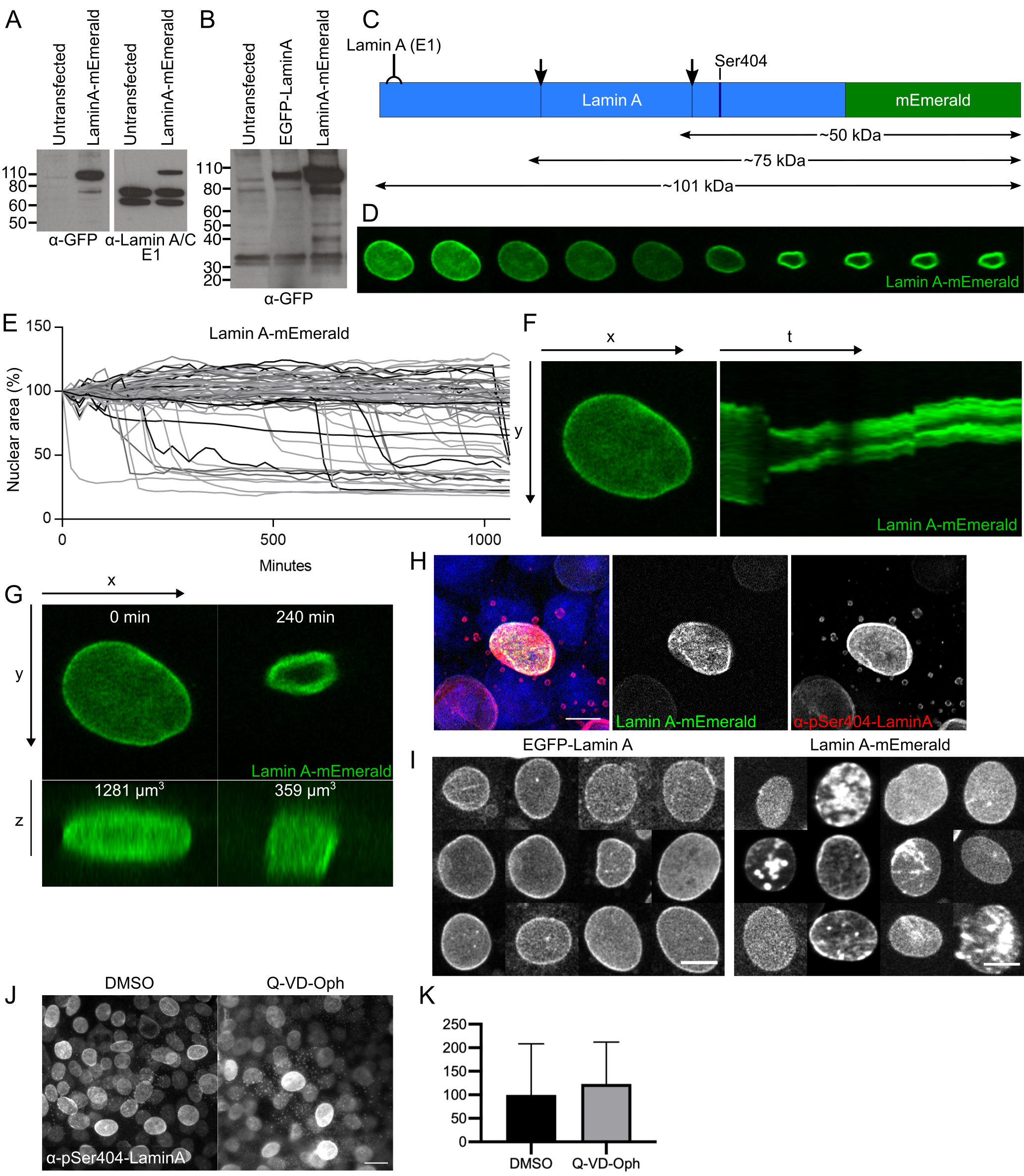

Supplement: Supplementary file 7 — Supplementary figure S4 [file 41418_2020_712_MOESM7_ESM.png]
