# Supplementary material for: Akt1-associated actomyosin remodelling is required for nuclear lamina dispersal and nuclear shrinkage in epidermal terminal differentiation
Source: Cell Death Differ. 2021 Jan 18;28(6):1849–64. doi: 10.1038/s41418-020-00712-9 (PMC8184862; doi:10.1038/s41418-020-00712-9)
Supplement: Supplementary file 2 — Supplementary Figure S1 Legend [file 41418_2020_712_MOESM2_ESM.docx]

**Supplementary Figure 1 – pSer404 Lamin A dispersal and loricrin expression in REKs.**

A – Human epidermal sections stained for pSer404 Lamin A/C.

B – Post-confluent REK cultures, untreated or expressing Myr-Akt1, stained for pSer404 Lamin A/C and Akt1.

C – Number of cells with dispersed pSer404 Lamin A/C in control or Myr-Akt1 expressing post-confluent REK cultures. 10 FOV per construct, unpaired t-test, ** p ≤ 0.01.

C – Number of cells with dispersed pSer404 Lamin A/C in control or Myr-Akt1 expressing post-confluent REK cultures. 10 FOV per construct, unpaired t-test, ** p ≤ 0.01.

E – Post-confluent REK cultures expressing WT, S404A or S404D Lamin A/C stained for loricrin. Scale bar = 100 µm.

F – Number of loricrin expressing cells in REK cultures expressing WT, S404A or S404D Lamin A/C. % of WT, > 3 FOV per experiment, one-way ANOVA, all comparisons non-significant.

G – Post-confluent REK cultures expressing WT or S404D Lamin A/C stained for pSer404 Lamin A/C. Scale bar = 50 µm.

H – Number of cells with dispersed pSer404 Lamin A/C in post-confluent REK cultures expressing WT or S404D Lamin A/C. % of WT, 3 FOV per construct, unpaired t-test, all comparisons non-significant.

I - Area of Hoechst 33342 staining of WT or S404D Lamin A/C expressing REKs.

J – Post-confluent REK cultures expressing WT, S404A or S404D Lamin A/C stained for FLAG. Scale bar = 100 µm.
