# Supplementary material for: Akt1-associated actomyosin remodelling is required for nuclear lamina dispersal and nuclear shrinkage in epidermal terminal differentiation
Source: Cell Death Differ. 2021 Jan 18;28(6):1849–64. doi: 10.1038/s41418-020-00712-9 (PMC8184862; doi:10.1038/s41418-020-00712-9)
Supplement: Supplementary file 4 — Supplementary Figure S2 Legend [file 41418_2020_712_MOESM4_ESM.docx]

**Supplementary Figure 2 –Akt1 interactor identification and inhibition of cytoskeletal candidates is required for nuclear size.**

A – Area of Hoechst 33342 signal of DMSO, blebbistatin and latrunculin B treated REKs, replicate experiment.

B - Area of Hoechst 33342 signal of DMSO, blebbistatin and latrunculin B treated REKs, replicate experiment.

C - Area of Hoechst 33342 signal of DMSO and CK666 treated REK cultures, replicate experiment.
