# Supplementary material for: Akt1-associated actomyosin remodelling is required for nuclear lamina dispersal and nuclear shrinkage in epidermal terminal differentiation
Source: Cell Death Differ. 2021 Jan 18;28(6):1849–64. doi: 10.1038/s41418-020-00712-9 (PMC8184862; doi:10.1038/s41418-020-00712-9)
Supplement: Supplementary file 6 — Supplementary Figure S3 Legend [file 41418_2020_712_MOESM6_ESM.docx]

**Supplementary Figure 3 – Live imaging of nuclei positive for Histone H2B-mCherry alone and with EGFP-Lamin A.**

A - Cross-sectional area over time of a further 44 Histone H2B-mCherry positive nuclei greater than 80 µm^2^.

B – Number of Histone H2B-mCherry positive nuclei that shrink and fade during imaging.

C - Cross-sectional area over time of Histone H2B-mCherry positive nuclei less than 80 µm^2^.

D - Cross-sectional area over time of Histone H2B-mCherry and EGFP-Lamin A positive nuclei.

E - Images every 20 min of a Histone H2B-mCherry and EGFP-Lamin A positive nucleus in post-confluent REKs.

F – Kymograph (yt) of a Histone H2B-mCherry and EGFP-Lamin A expressing nucleus.
