# Supplementary material for: Akt1-associated actomyosin remodelling is required for nuclear lamina dispersal and nuclear shrinkage in epidermal terminal differentiation
Source: Cell Death Differ. 2021 Jan 18;28(6):1849–64. doi: 10.1038/s41418-020-00712-9 (PMC8184862; doi:10.1038/s41418-020-00712-9)
Supplement: Supplementary file 8 — Supplementary Figure S4 Legend [file 41418_2020_712_MOESM8_ESM.docx]

**Supplementary Figure 4 – GFP-tagged Lamin A dynamics in post-confluent REKs.**

A – Untransfected or Lamin A-mEmerald expressing post-confluent REK lysates immunoblotted for GFP or the N-terminus of Lamin A/C (E1).

B – Overexposed blot of untransfected, EGFP-Lamin A or Lamin A-mEmerald expressing post-confluent REK lysates immunoblotted for GFP.

C - Diagram of Lamin A primary protein structure, highlighting predicted cleavage sites (arrows), Ser404, N-terminal antigen for antibody (E1), relative to N-terminal or C-terminal tags.

D - Images every 20 min of a Lamin A-mEmerald positive nucleus.

E - Cross-sectional area over time of Lamin A-mEmerald positive nuclei.

F – Kymograph (yt) of a Lamin A-mEmerald positive nucleus.

G - Xz projections of a Lamin A-mEmerald positive nucleus. Labelled with nuclear volume.

H – Lamin A-mEmerald positive REKs stained for pSer404 Lamin A. Confocal z-section, scale bar = 10 µm.

I – Morphology of EGFP-Lamin A and Lamin A-mEmerald positive nuclei in the first 30 min of imaging, 12 nuclei randomly selected from 12 FOV. Sum of confocal z-sections, scale bar = 10 µm.

J – Post-confluent REK cultures treated with DMSO or caspase inhibitor Q-VD-Oph stained for pSer404 Lamin A/C. Scale bar = 20 µm.

K – Number of cells with dispersed pSer404 Lamin A/C in post-confluent REK cultures treated with DMSO or Q-VD-Oph. % of DMSO, >10 FOV per construct, unpaired t-test, non-significant.
